# Films Based on Chitosan/Konjac Glucomannan Blend Containing Resveratrol for Potential Skin Application

**DOI:** 10.3390/ma18020457

**Published:** 2025-01-20

**Authors:** Karolina Kulka-Kamińska, Marzanna Kurzawa, Alina Sionkowska

**Affiliations:** 1 Department of Biomaterials and Cosmetic Chemistry, Faculty of Chemistry, Nicolaus Copernicus University in Torun, 7 Gagarin Street, 87-100 Torun, Poland; 2 Department of Analytical Chemistry and Applied Spectroscopy, Faculty of Chemistry, Nicolaus Copernicus University in Torun, 7 Gagarin Street, 87-100 Torun, Poland; jmk@umk.pl

**Keywords:** chitosan, konjac glucomannan, resveratrol, thin films, biomaterials

## Abstract

Biopolymers represent a significant class of materials with potential applications in skin care due to their beneficial properties. Resveratrol is a natural substance that exhibits a range of biological activities, including the scavenging of free radicals and anti-inflammatory and anti-aging effects. In this study, chitosan/konjac glucomannan resveratrol-enriched thin films were prepared. The enrichment of biomaterials with active ingredients is a common practice, as it allows the desired properties to be obtained in the final product. To characterize the films, several analyses were performed, including infrared spectroscopy, imaging of the samples by SEM and AFM techniques, swelling analysis in pH 5.5 and 7.4, mechanical and antioxidant assays, contact angle measurements, and determination of the resveratrol release profile under the skin mimicking conditions. Resveratrol incorporation into the matrices resulted in modifications to the chemical structure and film morphology. The mechanical characteristics of films with additives were found to undergo deterioration. The sample containing 10% of resveratrol exhibited a higher swelling degree than other films. The resveratrol-modified films demonstrated a notable antioxidant capacity, a reduced contact angle, and enhanced wettability. The resveratrol release occurred rapidly initially, with a maximum of 84% and 56% of the substance released depending on the sample type. Thus, the proposed formulations have promising properties, in particular good swelling capacity, high antioxidant potential, and improved wettability, and may serve as skin dressings after further investigation.

## 1. Introduction

Biomaterials can be defined as materials that are designed to interact with biological systems in terms of diagnosis, treatment, and/or replacement of various tissues and organs [1,2,3]. The history of biomaterials is as long as the history of humanity itself, as people have always employed a variety of materials to facilitate the healing of wounds or to replace damaged body parts [4]. In recent decades, there have been notable advancements in the field of biomaterials, encompassing both medical devices and tissue engineering [2,5]. The development of new technologies and the creation of new combinations of materials are occurring in response to the increasing demands of this field [6]. The increase in average life expectancy has had an impact on this. This positive trend has a number of consequences, including an increase in the number of procedures that save lives, help maintain health, or improve quality of life [6]. Biomaterials of all kinds are frequently employed for this purpose. These materials are required to demonstrate excellent biocompatibility, which necessitates their compatibility with blood, the absence of an inflammatory response or pyrogenic effects, the prevention of allergic reactions, and the lack of carcinogenic and toxic properties unless the objective is to target cancer cells [1,4,6]. The design and comprehensive testing of such a material necessitates the collaboration of scientists from a multitude of disciplines, including chemistry, biology, medicine, engineering, and materials science [1,6].

The skin is a vital organ with a plethora of functions, including thermoregulation, secretion, sensory function, protection against micro-organisms, and contribution to the body’s homeostasis [7,8]. It is the most externally located organ and is, therefore, the most susceptible to environmental factors: ultraviolet radiation, thermal, mechanical, or chemical agents. Given its extensive surface area, the likelihood of damage is increased. A disruption in the continuity of the skin or the skin and deeper tissues is defined as a wound [8]. There are multiple classifications of wounds. One such category encompasses both acute and chronic wounds, which are distinguished by differences in their healing duration, pathogenesis, and the health consequences they entail [8,9,10]. It is of great importance to consider the immune response, which, in the case of acute wounds, activates inflammation, promotes wound debridement, and accelerates healing [10]. Conversely, the impairment of this mechanism results in chronic inflammation, delayed healing, and the formation of chronic wounds [10]. Chronic wounds represent a significant economic burden for healthcare systems in many countries [8,10,11], with a particularly detrimental impact on patients’ quality of life [12,13]. It is estimated that between one and two percent of the population of developed countries is affected by this problem [14]. In general, any type of wound, including those that are uncomplicated, requires appropriate care and maintenance, which is related to the prevention of scar formation [11]. The management of skin damage is dependent on the type, depth, and surface area involved. The most appropriate method of treatment is dependent on the circumstances and may include the use of a dressing or tissue engineering techniques [14,15]. The purpose of a wound dressing is to provide optimal conditions for the healing process by covering the wound, while, in the field of tissue engineering, it is of paramount importance to utilize functional constructs that provide biological support to damaged tissue, thereby facilitating the processes of reconstruction and regeneration [14,15]. The second option represents a potential solution for patients with difficult-to-heal wounds and chronic wounds. The development of biomaterials for the promotion of dermal health based on biopolymers represents a broad avenue of research [16,17,18,19,20,21,22,23].

Biopolymers are macromolecules of natural origin that exhibit a number of beneficial properties, including biocompatibility, bioresorptivity, biodegradability, and bioactivity [16,24,25,26,27]. The aforementioned applications allow for the utilization of these materials in a variety of forms, including hydrogel, film, sponge, fiber, hydrocolloid, foam, and scaffold [9,20]. It has been demonstrated that these natural materials have the capacity to substitute for the extracellular matrix (ECM) and the skin’s cellular environment [20,28]. The second group of materials employed in the process of wound healing that merits mention is constituted by synthetic polymers. Synthetic polymers have been shown to possess a number of advantageous properties. These include low acquisition costs, stability, enhanced mechanical properties, and a wide variety of other properties [29,30]. However, synthetic polymer-based biomaterials are not inherently biodegradable and may result in the release of toxic by-products. Moreover, such materials frequently demonstrate an absence of positive biological activity [29]. To overcome the limitations of both material types, they are often combined [28,30]. In the domain of wound care, a diverse array of biopolymers is employed in the fabrication of dressings, encompassing both protein and polysaccharide polymers. These natural polymers exhibit distinctive characteristics, with each contributing to the healing process in a unique manner. Collagen, a prominent protein biopolymer, exerts a multifaceted influence on various stages of the healing process, including attracting fibroblasts to the wound site to stimulate proliferation, differentiation, and ECM production [28,31]. Historically, collagen was extracted from mammalian tissues, including pig hides, rat tails, and bovine tissues. However, due to concerns regarding the potential transmission of zoonotic diseases, the risk of immune reactions, and ethical considerations, the use of collagen from these sources has been limited [32,33]. A solution that has gained widespread acceptance and is devoid of the aforementioned issues is the extraction of collagen from marine sources, such as fish or sponges [32,33,34,35,36]. Other valuable polypeptides include silk, fibrin, elastin, and keratin [28]. Polysaccharides represent a diverse group of compounds that also find application in dermatological treatments. The presence of hydrophilic groups within their structure predisposes them to a wide range of modifications and additionally offers the possibility of interaction with the target site, which promotes adhesion [37]. A further advantage of this group is non-immunogenicity [38] or immunomodulatory [39]. In addition, polysaccharides of marine origins, such as fucoidan, hyaluronic acid, carrageenan, alginate, agar, and chitosan, are of great interest [37,39].

Chitosan (CS) is a chitin derivative that is extensively utilized in the field of medical sciences, largely due to its distinctive characteristics [40,41,42]. This polysaccharide consisting of D-glucosamine and N-acetyl-D-glucosamine units exhibits antibacterial activity, antioxidant capacity, and mucoadhesive properties [40,43,44,45]. The compound has a polycationic nature that positively influences its interactions with negatively charged groups in biological tissues, including the skin, mucous membranes, blood, and also bacterial cells [46,47,48]. CS is soluble in acidic solutions, which facilitate the protonation of amine groups [44,47,49]. The presence of hydroxyl and amino groups renders the substance susceptible to modifications [44,50,51]. The chemical modifications of functional groups facilitate the enhancement of the frequently unsatisfactory mechanical properties of chitosan materials [41,44,47]. Konjac glucomannan (KGM) is another biopolymer that possesses valuable properties. This polysaccharide is composed of D-glucose and D-mannose monomers, with approximately 8% of the main chain containing acetyl groups [52,53,54]. It is a water-soluble polymer derived from the tubers of the *Amorphophallus konjac* plant [54]. The material displays an excellent capacity to absorb water [55,56]. This substance is primarily known as a dietary fiber and is also utilized in traditional Chinese medicine for its beneficial effects on health, including the enhancement of immune functions, reduction in cholesterol levels, control of blood sugar levels, and improvement of vitamin B_6_ metabolism [52,53,57,58]. Nevertheless, the applications of konjac glucomannan extend well beyond the food industry. It is a polysaccharide employed in the development of drug delivery systems, the production of membranes, the manufacture of cosmetics, and the creation of wound dressings [55,59,60]. A range of studies has demonstrated the protective and regenerative potential of KGM for skin cells [61,62,63,64]. The enrichment of polymer matrices with active ingredients is a common practice employed in the development of materials with defined properties.

A compound with multifunctional properties and proven health-promoting effects is resveratrol, a stilbene derivative [65,66]. The synthesis of these compounds represents a defensive strategy deployed by plants in response to environmental stressors, including fungal infections and physical trauma [65,67,68]. This compound is found in a multitude of plant species, including the most prevalent source, grapes, as well as cranberries, blueberries, peanuts, and Japanese knotweed (*Reynoutria japonica)* [68,69]. Resveratrol exists in two forms, designated as *trans* and *cis* isomers. The *trans* isomer is a naturally occurring form that exhibits biological activities. These include remarkable antioxidant capacity, cardioprotective effects, anti-aging properties, anti-inflammatory activity, and anti-cancer effects [65,67,69]. The *trans* isomer can be transformed into the *cis* form due to several factors, including exposure to ultraviolet radiation, the process of fermentation, or the presence of a high pH [68,69]. The three hydroxyl groups present in resveratrol are involved in a number of important processes, including the scavenging of free radicals, the formation of metal complexes, and interactions with larger molecules [66]. Topically applied, it has a beneficial effect on the regenerative processes occurring in damaged skin tissue. This is due to its ability to regulate angiogenesis in wounds, protect and stabilize cell proliferation through the reduction in oxidative stress, and prevent scar formation [70]. This makes resveratrol an effective ingredient for use in a wide range of biomaterials.

The objective of the present study was to obtain polymer films based on a mixture of CS and KGM enriched with resveratrol. As demonstrated in a previous study, chitosan and konjac glucomannan are well-miscible polymers, and film materials based on this combination exhibit favorable properties [71]. We assume that the incorporation of resveratrol into the polymer matrix described herein will enhance the physicochemical characteristics of the resulting film material while simultaneously imparting antioxidant properties, which will be beneficial, especially in cutaneous topical applications. In addition, resveratrol, being a lipophilic substance, is considered a good candidate for facilitated penetration through the stratum corneum and the lipid bilayer of cells [72,73], as well as a substance that stimulates collagen production and the synthesis of other components of the extracellular matrix [74,75]. However, these aforementioned aspects remain largely speculative within the context of this study and are not examined herein. The properties of resveratrol cited above served as a guideline for the selection of this substance for our applications. Moreover, the material’s antioxidant activity is crucial for the wound healing process, where oxidative stress can impede its course [76,77,78]. To the best of our knowledge, polymer films with such a matrix composition containing resveratrol have not been investigated. In this study, the aforementioned materials were characterized in terms of their physicochemical and mechanical properties, free radical scavenging tests were performed, and the release profile of resveratrol from the matrix was determined.

## 2. Materials and Methods

### 2.1. Materials

Low molecular weight polymers—chitosan (Mv = 7.31 × 10^5^ g/mol) and konjac glucomannan (Mv = 7.71 × 10^5^ g/mol), resveratrol, 2,2-diphenyl-1-picrylhydrazyl (DPPH), and 2,2-azino-bis(3-ethylbenzothiazoline-6-sulfonic acid) (ABTS) were purchased from POL-AURA (Dywity, Poland). The acetic acid used in the preparation of the polymer solutions was supplied by the POCH (Gliwice, Poland). Phosphate-Buffered Saline (PBS) solution utilized for the swelling analysis was prepared using tablets procured from Life Technologies LTD. (Renfrew, UK). Di-sodium hydrogen phosphate (Na_2_HPO_4_) was bought from Chempur (Piekary Śląskie, Poland). Sodium dihydrogen phosphate (NaH_2_PO_4_) was purchased from Merck (Darmstadt, Germany). Ethanol was procured from Stanlab (Lublin, Poland). Trolox (6-hydroxy-2,5,7,8-tetramethylchroman-2-carboxylic acid) was supplied by Sigma-Aldrich (Søborg, Denmark).

### 2.2. Preparation of Polymer Films

Chitosan (CS) and konjac glucomannan (KGM) were separately dissolved in 0.1 M acetic acid, yielding solutions with concentrations of 2% and 0.5%, respectively. Subsequently, the CS and KGM solutions were combined in the 80:20 ratio, in accordance with the methodology outlined in the previous publication [71].

Next, 25 g of blended polymer solution was transferred to a 10 × 10 cm square Petri dish. This one served as the control sample. Samples with the additive were obtained via the addition of an ethanolic solution of resveratrol to the initial blend solution. This was conducted in increments of either 10% (BR10) or 20% (BR20) of the substance per mass of the polymers. Following this, the polymer solutions with resveratrol were stirred continuously at room temperature for an hour and then poured onto Petri dishes and allowed to dry. The drying process took three days. In order to obtain all the films in this study, the process of solvent evaporation was employed at room temperature (20–22 °C), without access to light.

### 2.3. Fourier Transform Infrared Spectroscopy (FTIR)

Infrared spectra were recorded using a Nicolet iS10 spectrophotometer, which was equipped with a diamond ATR attachment (Thermo Fisher Scientific, Waltham, MA, USA). A resolution of 4 cm^−1^ with a 64 times scan mode per sample was utilized. The examination of the samples was conducted within a wavelength range of 4000 to 400 cm^−1^. The data were analyzed and processed using the OMNIC (version 9.2.86) and Excel software (version 2409).

### 2.4. Scanning Electron Microscopy (SEM)

The surface of the films was imaged using a scanning electron microscope (Quanta 3D FEG, D9399, FEI, Eindhoven, The Netherlands). The films shown below were magnified 150 times. Preparation of the samples for analysis included coating with gold in order to provide a conductive surface for electron beam interaction.

### 2.5. Atomic Force Microscopy (AFM)

The roughness of the samples and their topography were examined using an atomic force microscope (MultiMode Scanning probe microscope, the NanoScope IIIa, Digital Instruments Veeco Metrology Group, Santa Barbara, CA, USA). The tapping mode of the apparatus was implemented at room temperature and air conditions. Gwyddion software (version 2.62) was used for the roughness designation from 5 μm × 5 μm AFM images.

### 2.6. Mechanical Properties

The films were cut into paddle shapes approximately 4 mm wide. They were then subjected to tensile tests on a mechanical testing machine (Z.05, Zwick and Roell, Ulm, Germany) under room conditions. The values of the test parameters such as initial force, crosshead speed fixed, and speed starting position were 0.1 MPa, 5 mm/min, and 50 mm/min, respectively. The test was employed to determine the values of Young’s modulus, tensile strength, and elongation at break. The results were collated using TestXpert II 2017 software. A one-way analysis of variance (ANOVA) test was employed to ascertain statistically significant differences between the samples.

### 2.7. Swelling Properties

The swelling analysis was conducted in two phosphate buffer solutions with pH values of 7.4 and 5.5, respectively. The films were cut into shapes of similar dimensions and mass and subsequently subjected to drying at a temperature of 45 °C for a period of 24 h. The samples were placed in dedicated containers with buffer solutions at 37 °C and their masses were tested after 15 min, 1 h, 2 h, 4 h, 8 h, 24 h, 48 h, and 72 h. Each time a sample was removed from the buffer and gently dried with a paper towel prior to measurement. A total of five samples of each type were subjected to testing, and the resulting data are presented as mean values with standard deviations. The following equation was used to calculate the swelling degree:

(1) $$Swelling=\frac{\left(m_{t}-m_{0}\right)}{m_{0}}\times 100\%\;[\%]$$

*m_t_*—the weight of the material after immersion in PBS [g]; *m*_0_—the initial weight of the material [g].

### 2.8. Contact Angle and Surface Energy

The analysis was conducted utilizing a goniometer equipped with a drop shape analysis system (DSA 10 produced by Kruss, Hamburg, Germany). The contact angle values of glycerin (G) and diiodomethane (D) were determined for each sample at a constant temperature. The data were presented as a mean value with a standard deviation. The Q-Dixon test was employed for the detection and elimination of outliers. The outcome of the contact angle analysis enabled the calculation of surface free energy (*γ_s_*), as well as, its polar (*γ_sp_*) and dispersive (*γ_sd_*) components in accordance with the Owens–Wendt method using Drop Shape Analysis (version 1.65.0.49) software.

The surface free energy and its polar and dispersive components were calculated using the Owen–Wendt method and the formula below [79]:

(2) $$\gamma_{L}*\frac{\left(1+cos\theta \right)}{2}={\left(\gamma_{s}^{d}*\gamma_{L}^{d}\right)}^{\frac{1}{2}}+{\left(\gamma_{s}^{p}*\gamma_{L}^{p}\right)}^{\frac{1}{2}}$$

$\gamma_{s}^{p}$—polar component of surface free energy; $\gamma_{L}^{d}$—dispersive component of surface free energy; *S*—solid substrate; *L*—liquid.

### 2.9. Moisture Vapor Transmission Rate (MVTR)

Samples with a diameter of Ø 33 mm were cut from the resulting films. Subsequently, the film sections were positioned on a dish containing 10 mL of distilled water. The dish containing the applied film was fixed in place with a ring of Ø 30 mm and additionally sealed with parafilm. The prepared dish was then weighed (*m*_1_) and subsequently placed in an incubator set at a temperature of 37 °C. A further measurement (*m*_2_) was taken after 72 h. The methodology described was adapted from a study conducted by Minsart et al. [80]. The following formula was employed to calculate the water permeation through the samples:

(3) $$MVTR=\frac{m_{1\;}-m_{2}}{t\;\times A}\;\left[g/24h\times m^{2}\right]$$

t—times [24 h]; A—area of water evaporation [m^2^].

### 2.10. Antioxidant Capacity

The antioxidant potential of films containing resveratrol was determined using DPPH (1,1-diphenyl-2-picrylhydrazyl) and ABTS (2,2-azino-bis(3-ethylbenzothiazoline-6-sulfonic acid)) Radical Scavenging Assays. The measurements described in the following section were made with a Shimadzu UV-1601 UV-Vis spectrophotometer (Kyoto, Japan). The calibration curves for the two methods were constructed with different concentration values. The different Trolox concentrations used in the ABTS and DPPH methods are due to the different reaction characteristics of the two methods and differences in their sensitivity and reactivity with different types of antioxidants.

#### 2.10.1. DPPH Radical Scavenging Assay

An ethanolic solution of 2,2-diphenyl-1-picrylhydrazyl (DPPH) at a concentration of 0.304 mM and an ethanolic solution of 6-hydroxy-2,5,7,8-tetramethylchroman-2-carboxylic acid (Trolox) at a concentration of 100 μM were prepared.

##### Calibration Curve Preparation

The following concentrations of Trolox were prepared in 10 mL volumetric flasks: 0.00; 2.50; 7.51; 12.52; 17.53; 22.54; and 25.04 mg·mL^−1^. Then 1.5 mL of ethanol, 0.5 mL of DPPH solution, and 0.5 mL of each concentration of Trolox were added to the plastic cuvettes. The mixtures prepared in this way were mixed and left for 15 min in a dark place. Next, the absorbances of the solutions were measured at λ = 517 nm using ethanol employed as a reference.

##### Sample Preparation and Measurement

The film fragments of comparable weight (~0.1000 g) were transferred to 10 mL graduated flasks and made up to the mark with ethanol. The films were extracted for one hour by shaking. Subsequently, 0.5 mL of each obtained extract, 1.5 mL of ethanol and 0.5 mL of DPPH solution were added to the plastic cuvettes, mixed, and left in a dark place for 15 min. Absorbance measurements were conducted in a manner analogous to that employed for the calibration curve.

#### 2.10.2. ABTS Radical Scavenging Assay

An aqueous solution of 2,2-azino-bis(3-ethylbenzothiazoline-6-sulfonic) acid (ABTS) at a concentration of 7 mM was prepared and mixed with 2.45 mM potassium persulfate (K_2_S_2_O_8_) solution in a ratio 2:1, respectively. Subsequently, the mixture was incubated in the absence of light for a period of 16 h. Next, the mixture was diluted with water in order to obtain an absorbance value of approximately 1.0 at λ = 734 nm. Simultaneously, an ethanolic solution of 6-hydroxy-2,5,7,8-tetramethylchroman-2-carboxylic acid (Trolox) at a concentration of 5 mM was prepared.

##### Calibration Curve Preparation

The appropriate volumes of Trolox solution were added to the 10 mL volumetric flasks to obtain the following concentrations: 0.00, 12.40, 24.80, 62.00, 99.20, 148.80, 186.00, 223.20, and 248.00 mg·mL^−1^. Then, 0.1 mL of each concentration of Trolox and 3.9 mL of ABTS^•+^ solution were added to the plastic cuvettes. The mixtures prepared in this way were mixed and left for 10 min in a dark place. Next, the absorbances of the solutions were measured at λ = 734 nm using ethanol employed as a reference.

##### Sample Preparation and Measurement

The film fragments of comparable weight (~0.1000 g) were placed in 10 mL graduated flasks and made up to the mark with ethanol. The films were extracted for one hour by shaking. Subsequently, 0.1 mL of each obtained extract and 3.9 mL of ABTS^•+^ solution were added to the plastic cuvettes, mixed, and left in a dark place for 10 min. Absorbance measurements were conducted in a manner analogous to that employed for the calibration curve.

### 2.11. Resveratrol Release from Polymeric Matrix

The amount of resveratrol released from the films was determined via high-performance liquid chromatography (UHPLC Shimadzu, Kyoto, Japan) with column 250/4.6 Nucleoshel RP 18.5 _m at a wavelength of 306 nm. Shapes of 2 cm × 2 cm and approximately the same weight were cut from each resveratrol film. Each sample was placed in 50 mL of PBS solution (pH = 5.5) at 35.5 ± 1 °C and stirred on a magnetic stirrer. Then, 1.5 mL of solution was collected at the following intervals from the start of the analysis: 1; 3; 5; 10; 15; 30; 45; 60; 90, and 120 min. At each sample collection, the solution was replenished with 1.5 mL of fresh PBS. Three replicates were made for each sample type. The percentage release of resveratrol from the films was calculated using the following formula [81]:

(4) $$\%Res=\frac{M_{t}}{M_{0}}\times 100\%$$

*M_t_*—the amount of resveratrol released at different time points; *M*_0_—the amount of resveratrol in the film.

## 3. Results

### 3.1. Fourier Transform Infrared Spectroscopy (FTIR)

**Figure 1:**
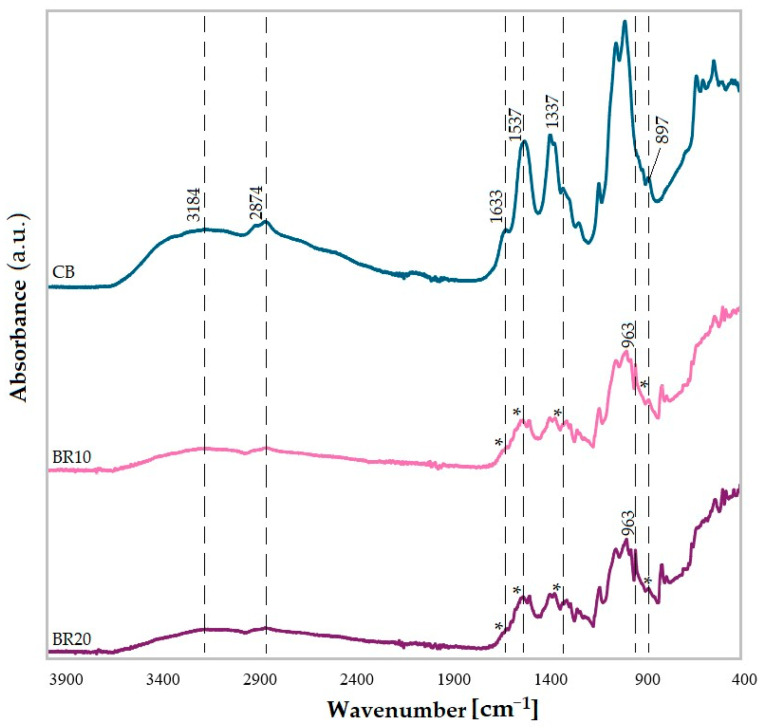
Infrared spectra of films: control blend (CB), blend with addition of 10% of resveratrol (BR10) and blend with addition of 20% of resveratrol (BR20). Asterisks (*) denote changes in comparison with the control sample.

The infrared spectra of the control sample (CB) exhibited the characteristic bands for the CS/KGM blend, as previously reported [71]. The broad band at 3184 cm^−1^ is indicative of O-H and N-H stretching vibrations, as well as the presence of hydrogen bonds between the two biopolymers [82]. The peak observed at 2874 cm^−1^ is indicative of the presence of C-H vibrations from both polysaccharides [83]. The bands appearing at 1633 cm^−1^, 1537 cm^−1^ and 1337 cm^−1^ can be attributed to the Amide I C=O stretching, Amide II N-H bending, and Amide III C-N stretching vibrations from chitosan, respectively [84,85,86]. Nevertheless, the band at approximately 1630 cm^−1^ can be linked to the O-H vibration mode, which is connected to the water content of the sample [87,88]. Furthermore, the bands at 1063 cm^−1^ and 1017 cm^−1^ were assigned to stretching vibrations of the C-O, C-H, and C-C ring [89,90,91]. The peaks at 1152 cm^−1^ and 897 cm^−1^ are characteristic of glycosidic bonds [89,92] (Figure 1).

The spectra of the films with resveratrol exhibit alterations in both wavelength and intensity when compared to the control sample. This indicates the potential for an interaction between the polymer matrix and the active substance. The spectra of the BR10 and BR20 films have an almost identical pattern. The absorption band at approximately 3184 cm^−1^, derived from the phenolic resveratrol’s O-H group [93], is overlapped by the O-H and N-H vibration signal of the chitosan/konjac blend. It can be observed that in both cases, the band at 1630 cm^−1^ has undergone a significant reduction in intensity, approaching complete disappearance. This may be related to the lower water content of the samples. Furthermore, an additional peak at 963 cm^−1^ is distinguishable, which can indicate the trans olefin bond in resveratrol. This confirms the presence of this compound in the films [93,94].

### 3.2. Scanning Electron Microscopy (SEM)

**Figure 2:**
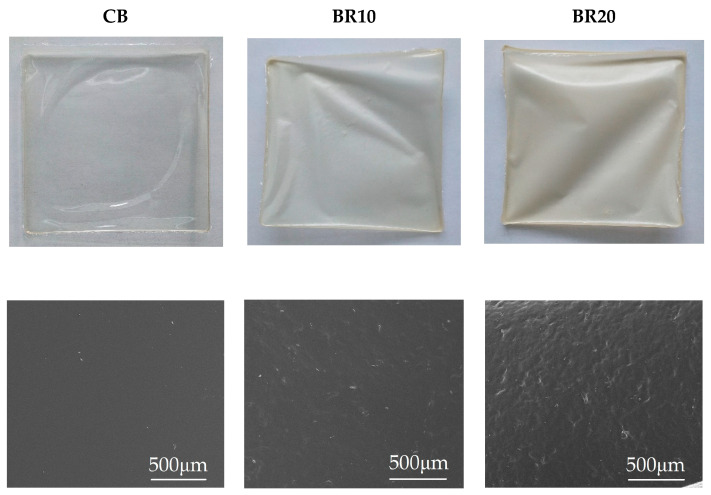
A compilation of photographs and SEM images for the samples tested: CB, BR10, and BR20.

Scanning electron microscopy (SEM) images provide insight into the surface characteristics of the polymer films. The pure film, based on the CS/KGM blend (CB), displays a uniform and smooth surface. The incorporation of resveratrol resulted in a reduction in the homogeneity of the material, accompanied by an increase in surface undulation. As the concentration of resveratrol in the sample increases, the film becomes increasingly opaque, as evidenced by the images presented in Figure 2.

### 3.3. Atomic Force Microscopy (AFM)

**Figure 3:**
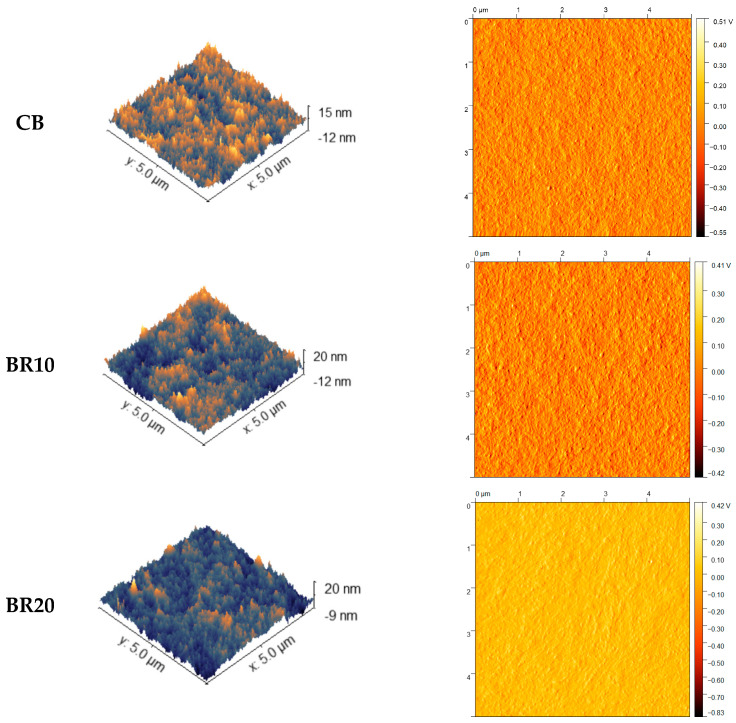
AFM visualization of CS/KGM films without (CB) and with resveratrol (BR10, BR20).

The topographical characteristics of the films were determined through an analysis of the AFM images (Figure 3). From these data, the roughness of the samples was determined. The roughness is directly correlated with the adhesion properties of the material and the interactions with the surface [95,96]. The incorporation of resveratrol into the polymer matrix at a concentration of 10% resulted in a slight increase in film roughness. An increase in the concentration of the substrate in the sample resulted in a roughness reduction, with the value lower than the initial film in the absence of the additive (Table 1). However, the topographic image of the surface of all the samples reveals the presence of numerous densely packed small rises, which may serve as potential contact points with the target surface.

### 3.4. Mechanical Properties

**Figure 4:**
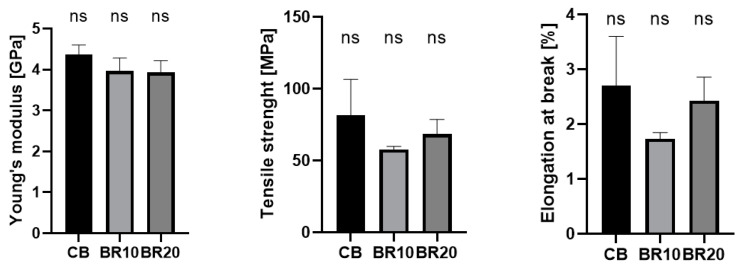
Mechanical properties of pure blend and resveratrol-incorporated blend films. The results were not statistically significant (ns).

The results of the mechanical tensile tests carried out on the obtained films are presented in Figure 4. The control sample exhibited the highest value for Young’s modulus. The addition of resveratrol resulted in a slight decrease in this parameter. A similar trend was observed in the tensile strength, where resveratrol incorporation led to a reduction in this value. The lowest tensile strength was observed for the BR10 sample. The elongation at the breaking point exhibited a similar trend. The results indicate that the addition of resveratrol weakened the mechanical properties of the material. This suggests that the structure of the material was modified, as evidenced by the infrared spectra.

The thickness of the films was summarized in Table 2. These values were collected prior to the performance of the mechanical tests. It has been observed that an increase in the amount of resveratrol results in a corresponding increase in the thickness of the formed film.

### 3.5. Swelling Properties

A swelling analysis was conducted in PBS solutions with two distinct pH values (pH = 7.4 and 5.5). The test was conducted for a period of up to 72 h. The swelling ability was observed in all samples, independently of the pH value of the solution.

**Figure 5:**
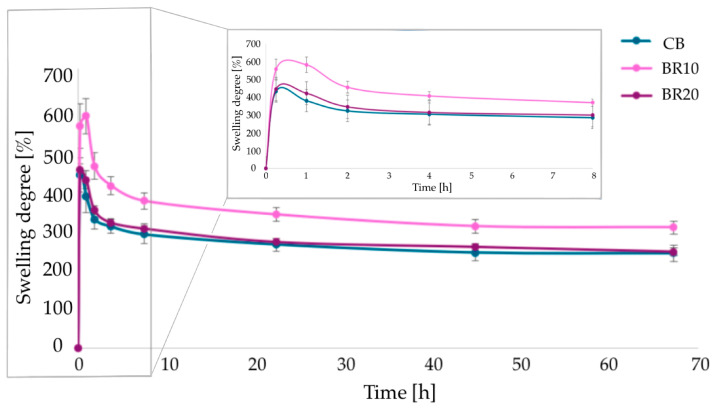
The results of the swelling analysis for the film materials carried out at pH = 7.4 (presented as mean values with standard deviation (SD)).

Polymer samples tested at pH 7.4 exhibited maximum swelling within the initial hour of analysis (Figure 5). Sample BR10 displayed the most pronounced degree of swelling, reaching a value of 581%. Sample BR20 exhibited a slightly higher percentage value in comparison to the pure CS/KGM film (CB). A further reduction in the weight of the samples suggests the commencement of degradation processes.

**Figure 6:**
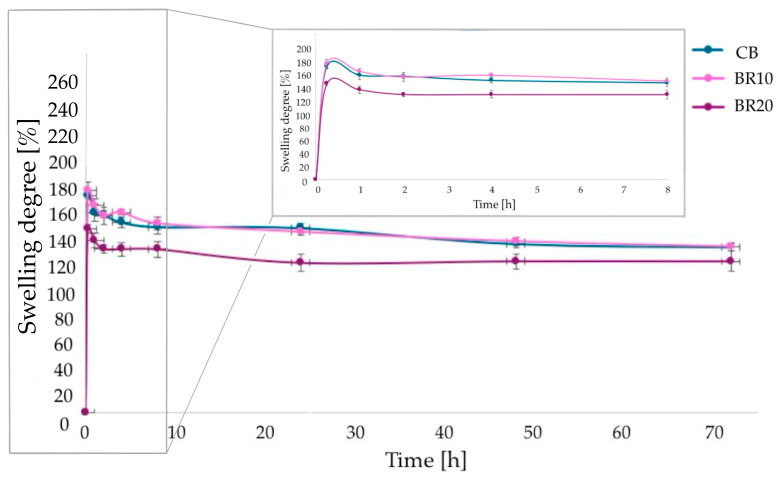
The results of the swelling analysis for the film materials conducted at pH = 5.5 (presented as mean values with standard deviation (SD)).

Samples tested in PBS solution at pH 5.5 showed a significantly lower swelling potential (Figure 6). Sample BR10 exhibited the highest swelling potential (180%), a value that is only slightly higher than that observed for CB (176%). Sample BR20 swelled the least (149%). Furthermore, the analysis exhibited a comparable trend to that observed at pH 7.4, with the highest values recorded during the initial hour of testing.

The swelling properties of the polymer sample can be explained by the arrangement of the polymer chains and the interactions that occur between the polymers (CS and KGM) and the additive. Changes in the arrangement of the polymer chains result in the modified density of the polymer sample; the lower the density of the polymer network, the higher the swelling capacity of the sample [97]. In the context of a slightly acidic environment characterized by a pH value below the pKa value of CS, the protonation of amine groups within the polymer results in a denser structure. In general, samples examined at pH 5.5 exhibited lower swelling behavior than at pH 7.4. In an almost neutral environment, the amine groups of CS are deprotonated, thereby exerting an influence on a chain structure that is more loosely arranged. Another factor that must be considered is the cross-linking of the polymer matrix, which affects the degree of swelling. The more cross-linking bonds, the less the chain can stretch and the less the sample swells [98]. The addition of resveratrol to the blend polymer matrices caused changes in chain conformation, which can be observed in swelling analysis. The incorporation of 10% of resveratrol into the polymer matrix probably resulted in a relaxation of the chains, thereby facilitating the absorption of water molecules. Similar conclusions were made by Pastor et al., where the authors suggested that resveratrol disrupts the arrangement of polymer chains, leading to the formation of a more disordered network [99].

### 3.6. Contact Angle and Surface Energy

**Figure 7:**
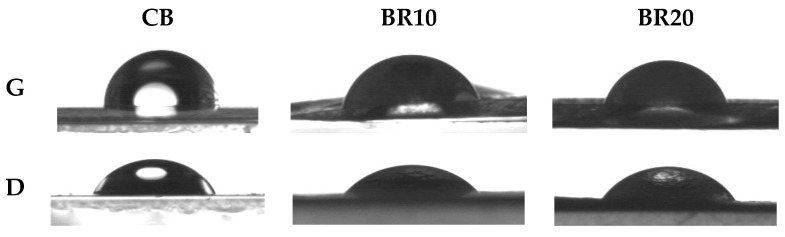
Images of drops during contact angle measurement (G—glycerin; D—diiodomethane).

Contact angle measurements using the sitting drop method allowed the wettability of the sample surfaces to be assessed. Figure 7 provides a visual documentation of the droplets captured during the analysis. The initial CB sample exhibited a wetting angle of approximately 102°, indicative of its hydrophobic nature. The addition of resveratrol to the matrix resulted in a reduction in the contact angle value and a notable enhancement in the wettability of the film. It was observed that as the concentration of resveratrol in the sample increased, the wetting angle decreased, indicating an improvement in surface wettability. The highest surface energy was observed for the sample BR10, which also exhibited a high value for its dispersion component (Table 3).

### 3.7. Moisture Vapor Transmission Rate (MVTR)

**Figure 8:**
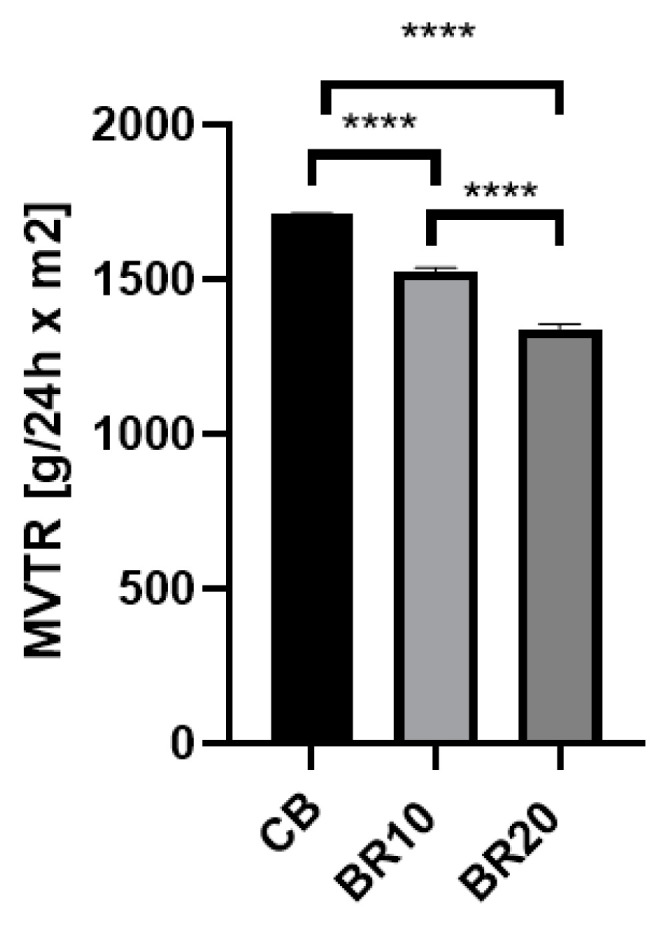
Moisture vapor transmission rate (MVTR) of the tested films. The results were statistically significant (**** p < 0.0001).

Moisture permeation through the material is important in skin applications [80]. The findings of the analysis indicated that the initial film, comprising CS and KGM (CB), exhibited the highest value for this parameter, reaching 1710 g/m^2^/24 h. For films with resveratrol, we observed a reduced value of this parameter, which was 1526 g/m^2^/24 h for sample BR10 and 1341 g/m^2^/24 h for sample BR20. It was observed that an increase in the concentration of the stilbene derivative within the material resulted in a notable reduction in moisture permeability (Figure 8).

### 3.8. Antioxidant Capacity

Resveratrol is recognized as a potent antioxidant with the capacity to scavenge free radicals to a remarkable extent [100]. The antioxidant activity of resveratrol-enriched polymer films was investigated using the DPPH and ABTS methods.

The antioxidant potential of resveratrol is associated with the high number of hydroxyl groups present in this molecule [100]. In addition, two structural adjustments of resveratrol are crucial for its radical scavenging activity: the para-hydroxyl group in the first ring, which possesses electron-donating properties, and the double bond between the rings, which enhances the delocalization of electrons [100]. The scavenging of radicals is dependent on factors such as the solvent and the radical character [101]. A number of studies have confirmed that the resveratrol radical scavenging properties are the result of SPLET (sequential proton-loss electron transfer) and HAT (hydrogen atom transfer) mechanisms [101,102,103]. In the context of the DPPH method, the antioxidant effect is contingent on the availability of hydrogen. In contrast, the ABTS method involves the scavenging of a proton radical through the transfer of electrons [104]. This discrepancy in sensitivity is likely the cause of the observed variations in results between the two methods.

#### 3.8.1. DPPH Radical Scavenging Assay

The DPPH method is a relatively simple method for testing the free radical scavenging activity of antioxidants. The solution of the DPPH radical exhibits an absorption maximum at 517 nm and displays a deep purple color, which gradually fades in accordance with the number of electrons that are taken up [105,106].

**Figure 9:**
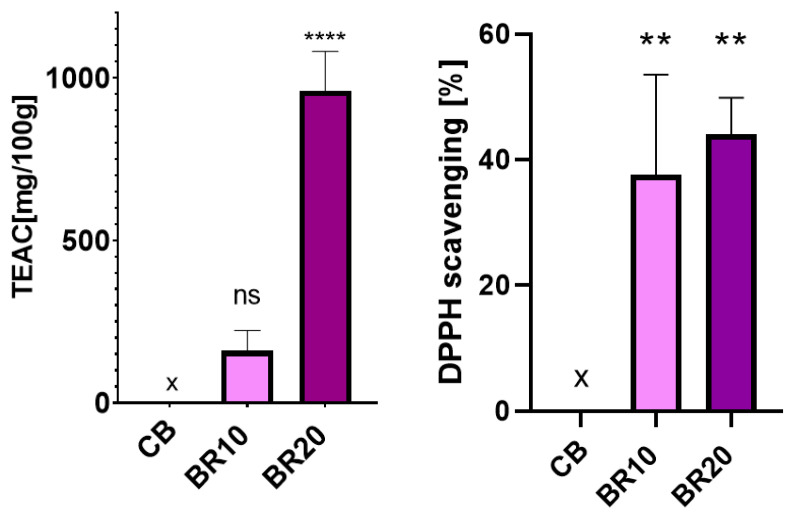
Antioxidant test results for films without (CB) and with resveratrol (BR10, BR20) obtained by the DPPH method. Data are presented as a mean value with a standard deviation. Statistically significant differences are indicated in comparison with the control sample (CB) as follows: ** *p* < 0.03; **** *p* < 0.0001; ns—not significant.

The outcomes of the DPPH test are illustrated in Figure 9. It can be observed that the initial film exhibited no antioxidant characteristics. As the resveratrol concentration in the remaining films increased, their antioxidant properties were also enhanced. The antioxidant capacity was expressed as a Trolox equivalent antioxidant capacity per 100 g of film sample and the percentage of DPPH radical inhibition.

#### 3.8.2. ABTS Radical Scavenging Assay

The ABTS method represents another simple, quick, and sensitive approach to the determination of antioxidant activity, utilizing spectrophotometric measurements. The degree of reduction in a dark green ABTS radical is quantified by measuring the absorbance at 734 nm upon reacting with an antioxidant [106].

**Figure 10:**
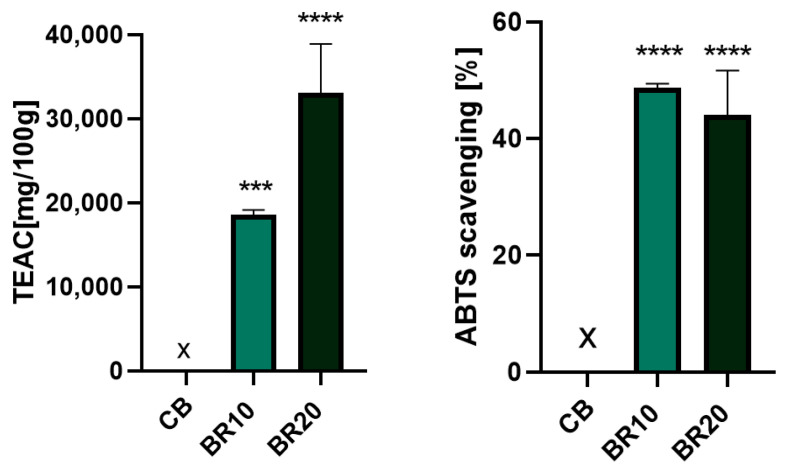
Antioxidant test results for films without (CB) and with resveratrol (BR10, BR20) obtained by the ABTS method. Data are presented as a mean value with a standard deviation. Statistically significant differences are indicated in comparison with the control sample (CB) as follows: *** *p* = 0.0009; **** *p* < 0.0001.

The results of the antioxidant assays conducted using the second method demonstrated that the films containing resveratrol exhibited high activity in neutralizing the ABTS radical (Figure 10). This finding aligns with the regularity observed in the DPPH method, whereby an increase in resveratrol content in the sample was associated with enhanced antioxidant activity. The results were also expressed as the Trolox equivalent and the percentage of ABTS radical inhibition. The graph displays the inhibition percentage, which is marginally lower for the BR20 sample in comparison with the BR10 film. However, it should be noted that this is a value calculated for a twice-diluted extract, as it had to be fitted to the calibration curve.

### 3.9. Resveratrol Release from Polymeric Matrix

**Figure 11:**
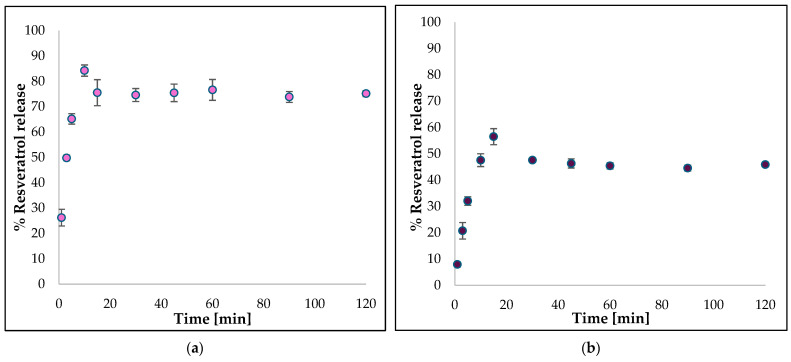
The release profile of resveratrol from the: (**a**) BR10 sample; (**b**) BR20 sample at 35.5 °C.

Resveratrol release was evaluated under conditions mimicking the skin environment in PBS solution at pH 5.5 and 35.5 °C (Figure 11). The active substance from both films was released within the first 10 to 15 min, exhibiting a rapid initial release, or “burst release”, as it is commonly termed. The maximum quantity of the compound released from film BR10 was 84%, while the maximum quantity released from film BR20 was 56%. The release profiles of the two samples exhibited near-identical characteristics. Following a 15-min analysis period, the release was observed to remain constant at approximately 75% and 45% for BR10 and BR20, respectively.

The observations pertaining to the release profile are complementary to the results of the swelling process. From sample BR10, a greater percentage of resveratrol was released than from sample BR20, which correlates with the swelling behavior of the polymer films in solution at pH 5.5. As previously mentioned, sample BR10 exhibited the highest degree of swelling, which may be attributed to the relatively loose arrangement of the chains in the polymer matrix, resulting in the facile release of the active substance. Conversely, sample BR20 exhibited a comparatively low degree of swelling, indicative of a more compact polymer structure, which in turn correlated with a more constrained release of resveratrol. Simultaneously, the release of the substance can be regulated by the concentration gradient between the polymer matrix and the solution into which it penetrates [107].

## 4. Discussion

Resveratrol is a natural compound with a wide range of potential applications [68]. It can be used internally, for example, in food or dietary supplements, where it has the potential to exert a beneficial effect on the whole body: anti-diabetic, anti-inflammatory, anti-tumor, and protective on the cardiovascular system [108,109]. When applied externally to the skin, resveratrol has been demonstrated to possess anti-aging, brightening, antioxidant, and anti-cancer properties [110]. Consequently, it can be incorporated into cosmetic preparations and medical products, where it may be employed in the treatment of skin cancer, melanogenesis disorders, allergies, and infections [75,110]. A study of the available literature and chemical properties indicates that resveratrol has the potential to penetrate the skin barrier [75].

When the skin barrier is damaged as a result of trauma, resveratrol has the potential to support the normal wound healing process by promoting fibroblast maturation, collagen deposition, and the formation of new blood vessels [111,112,113]. Dressing materials are becoming increasingly sophisticated, offering not only physical protection against contaminants and other harmful agents but also acting in a bioactive manner, releasing substances that promote the healing process [111,114].

Biopolymers offer a promising avenue of research in the field of dressings, given their aforementioned characteristics, including biocompatibility and, in many cases, bioactivity. CS is a biopolymer that has been extensively investigated for use in dressing materials, and products based on it have already been deployed in practice [115]. A review conducted in 2019 by Matica et al. [115] identified the presence of this material in over 20 dressing products on the market, which were observed in a range of forms, including sponge, gel, spray, and chitosan-soaked gauze. Additionally, KGM is a valuable natural polymer employed in skin products, including dressings, due to its notable biocompatibility and gelling properties [54,62,63]. However, due to the unsatisfactory mechanical characteristics of its base products, it is predominantly employed in conjunction with other polymers [54,61,116,117]. In the present study, a blend of CS and KGM was used as the starting point into which resveratrol was incorporated to obtain a thin film. The implementation of resveratrol into the blend resulted in alterations to the physicochemical properties of the film.

The addition of resveratrol resulted in a notable change in the morphology of the films, with a distinct increase in irregularity. Our findings align with those of Pastor et al. [99], who investigated the effects of resveratrol on chitosan and methylcellulose-based films at varying concentrations. Scanning electron microscopy images demonstrated the modifications in surface smoothness of the samples, exhibiting the irregularities of the surface as the concentration of stilbene derivative increased. The pure CS polymeric film displayed homogeneity and a smooth surface. The authors suggested that resveratrol disrupts the packaging of polymer chains, leading to their rearrangement and the formation of a more disordered network. Moreover, the opacity of the films increased in correlation with the elevated concentration of resveratrol present in the samples [99], this finding aligns with our observations. The topography of the surface was also examined in the aforementioned study by Pastor et al. [99]. The concentration of stilbene derivatives was identified as the key factor influencing roughness, with the highest content of this substance significantly increasing the roughness value [99]. Roughness is a significant factor in determining the contact area of material with other surfaces and is a crucial parameter in the context of adhesion properties [118,119]. It is evident that an increase in the roughness of the film results in an enlargement of the surface area, as well as the area of contact. The skin is a particularly complex object of study in relation to adhesion due to its intricate topography and the significant variability in its roughness that is observed depending on factors such as human age or body location [119].

The mechanical properties of films containing resveratrol have decreased; however, they are not critical from the usage and further analysis perspective. In a study conducted by Zhang et al. [120], polymeric films were prepared containing CS and an inclusion complex with resveratrol and beta-cyclodextrin. The tensile strength of the formulations was observed to decrease as the volume of the complex with resveratrol added increased [120]. In a study by Samprasit et al. [121], the properties of polyvinyl alcohol/chitosan films containing resveratrol and mangostin were assessed. The incorporation of active substances resulted in a reduction in the mechanical resistance of the samples. The tensile strength and elongation at break of the films containing active substances were found to be lower than those of the pure polymer samples [121]. Similar observations were made by Pastor et al. [99], where the mechanical parameters were found to be less favorable for the films with resveratrol content. It was observed that an elevated concentration of resveratrol in the sample resulted in a notable reduction in the film’s flexibility. The addition of resveratrol resulted in a reduction in both the elongation at break and tensile strength values of the films. The authors suggest that this phenomenon can be attributed to the reduction in intermolecular forces present in the polymer matrix in response to the addition of a non-miscible compound [99,122].

The degree of swelling is a crucial parameter for materials that may be used in applications involving direct contact with the skin [123]. In the present study, two solutions with varying pH values were employed to assess this parameter. The intent was to create a simulated environment of healthy skin by using phosphate-buffered saline (PBS) at a pH of 5.5, while a pH of 7.4 was used to create a simulated wound condition. Our research indicates that the films exhibit a diminished capacity to swell in a slightly acidic solution. Samprasit et al. [121] evaluated the swelling properties of chitosan-based films containing resveratrol at pH 5.5. The degree of swelling observed in the samples treated with resveratrol in the aforementioned study is comparable to that observed in our samples, reaching 123% and 142%, respectively. They also observed a correlation between the hydrophobicity of the films and their swelling behavior, noting that more hydrophobic films exhibited reduced swelling. The amino and hydroxyl groups present in the polymers are primarily responsible for the observed swelling properties, which are a result of their ability to interact with water through the formation of hydrogen bonds [121]. According to our study, at higher pH, which is characteristic of a wound, the degree of swelling is considerably higher. This is a beneficial property in the context of wound management, as it enables the collection of exudates from the wound site [124]. Gani et al. [125] examined the swelling behavior of materials based on nano CS containing resveratrol in different solvents. It was observed that an alkaline environment facilitates enhanced swelling [125]. It should be noted that the thickness of the polymer films can also have a direct impact on the results of swelling. In the present study, the thickness of the films was found to increase with the addition of resveratrol. Sample BR10 was found to be particularly thin, exhibiting a thickness that was very similar to that of the control sample. This sample also exhibited the highest swelling ability in both pH values. The BR20 sample exhibits both the highest thickness and the lowest swelling ability at pH values of 5.5 and 7.4. This finding aligns with the established principle that the depth of solvent penetration is reduced in thicker films when compared to thinner films [126].

There is no defined single right value for moisture transmission for dressing materials. Some studies provide a value less than 840 g/m^2^/24 h [127,128] as appropriate for supporting the healing or a range of 2000–2500 g/m^2^/24 h [80]; however, the specific wound type is the most relevant factor in determining the appropriate application [127,128]. In the case of wounds that exhibit high exudate, such as burns, the utilization of a dressing with elevated water permeability is essential. Conversely, for wounds presenting as dry, the employment of materials characterized by exceptionally high MVTR is not requisite. The polymeric films fabricated in this study exhibited relatively high MVTR values. However, the incorporation of resveratrol resulted in a decrease in this parameter. The observations made in this study are consistent with those reported by Pastor et al. [99], who found that a stilbene derivative reduced the water permeability of polymeric films composed of methylcellulose or CS [99]. A factor influencing the moisture permeability of a polymeric material may be the density of the structure. As shown in the SEM images, all polymer films studied were characterized by a rather compact, dense surface structure. Increased density may limit the permeability of the polymer material [129]. Another factor affecting the limited permeability may be the nature of the substance incorporated into the film [129]. Resveratrol, a substance with only slight water solubility within the polymer film, may impede the permeation of water molecules through the film.

Antioxidant tests served to confirm the well-known properties of resveratrol, which has been established as an effective free radical scavenger. This compound was found to enrich polymeric materials with antioxidant properties that were not previously evident. The utilization of antioxidant materials in the context of wound dressings is a desirable practice, as these materials have been shown to promote healing through active mechanisms. Antioxidant dressings help to maintain an environment conducive to healing and restore balance in cases of oxidative stress and increased metalloproteinase activity [76]. Excessive levels of reactive oxygen species (ROS) have been demonstrated to induce prolonged inflammation, which in turn has been shown to delay the healing process and result in chronic wound formation [76,130]. Conversely, low levels of oxidative forms have been shown to facilitate wound closure through the stimulation of angiogenesis and the promotion of re-epithelialization [130]. Therefore, it is essential to maintain optimal levels of oxidative species to ensure optimal wound healing. In a study by Pastor et al., where a DPPH test was performed, it was noted that the antioxidant efficacy of resveratrol was high and practically unchanged during film formation, drying, and conditioning [99].

The release profile of the substance from the material provides significant information regarding the material’s effectiveness and facilitates the estimation of its potential applications. It is important to note that a number of factors can affect the release, including the degree of swelling or roughness of the films. In a study by Samprasit et al. [121], the release profile of resveratrol from a CS matrix was evaluated. The study revealed a correlation between the release of the substance and both the swelling and the mechanical properties of the material. The higher swelling ability was connected to the better diffusion and release of the active substance [121]. In the present experiment, the highest percentage of released resveratrol was observed in the sample with the lowest concentration of the substance. The highest swelling degree was exhibited by the same film at pH 5.5. This finding aligns with the conclusions put forward in the aforementioned study [121]. In addition, Zemljic et al. have indicated the role of swelling in diffusion-controlled processes during the release of the substance [123]. The release of substance is also influenced by the roughness of the material. Greater roughness is hypothesized to result in enhanced permeation of the substance from the film [131]. This finding aligns with the experimental observations reported in our research, wherein the BR10 sample, characterized by its elevated roughness, exhibited the optimal release capacity for resveratrol. Furthermore, Ansari et al. evaluated the release of resveratrol from a CS/ethylcellulose matrix. They observed that an enhancement in the release of the drug was concomitant with an increase in the hydrophilic polymer content [132]. Another factor that merits mention is the structure of the samples, which has been shown to influence the release characteristics. In a study by Boateng et al., films with dense characteristics and porous structures were compared in terms of active substance release. The researchers noted that porous materials exhibited faster release of the substance [133]. SEM images of all films presented their rather dense structures. In summary, as previously stated, the active ingredient was released from both films at a relatively rapid rate in the present study, which is advantageous for cosmetic applications. Cosmetic products, such as sheet masks, are not retained on the skin for extended periods, and the efficacy of the active ingredient is of paramount importance. For medical applications, such as wound dressings, this may also be applicable due to the rapid prevention of infection and the immediate anti-inflammatory effect.

## 5. Conclusions

The CS/KGM resveratrol-enriched thin films were successfully prepared. The incorporation of resveratrol into the biopolymeric matrices led to the modification of the film’s morphology and mechanical properties. Furthermore, the stilbene derivative addition to the CS/KGM blend resulted in a disruption of the arrangement of the polymer chains, leading to their reorganization. This, in turn, particularly translated into the films’ ability to swell and release the substance. The BR10 sample demonstrated exceptional properties across the majority of the analyses conducted. The resveratrol-modified films demonstrated a notable antioxidant capacity, a reduced contact angle, and enhanced wettability. The resveratrol release from biopolymeric matrices occurred rapidly initially, with a maximum of 84% and 56% of the substance released, depending on the sample type. The high release of the active substance, the high antioxidant activity, the swelling ability, and the favorable wetting properties of the materials show their potential to be used as products for skin applications. The proposed formulations have promising properties and, after further investigation, may serve as skin dressings.

## Figures and Tables

**Table 1:** Roughness parameters for tested films.

Sample	R_q_ [nm]	R_a_ [nm]
CB	3.13 ± 0.03	2.49 ± 0.01
BR10	3.81 ± 0.49	3.03 ± 0.39
BR20	2.85 ± 0.23	2.24 ± 0.20

**Table 2:** The thickness of tested polymer films.

Sample	Thickness [mm]
CB	0.029 ± 0.003
BR10	0.031 ± 0.006
BR20	0.036 ± 0.004

**Table 3:** Summary of contact angle and surface free energy results for individual samples.

Specimen	Θ^G^	Θ^D^	γ_s_ [mJ/m^2^]	γ_s_^d^ [mJ/m^2^]	γ_s_^p^ [mJ/m^2^]
CB	101.87 ± 2.90	67.9 ± 3.43	24.29 ± 1.80	24.15 ± 1.71	0.13 ± 0.08
BR10	77.78 ± 2.87	45.7 ± 7.66	36.16 ± 3.95	32.92 ± 3.99	3.24 ± 0.05
BR20	73.46 ± 4.18	51.48 ± 4.96	34.29 ± 3.09	28.25 ± 2.12	6.04 ± 0.97

## Data Availability

The original contributions presented in the study are included in the article; further inquiries can be directed to the corresponding author.
